# Range extension and first records of *Coryphaenoides striaturus* Barnard, 1925 and *Coryphaenoides subserrulatus* Makushok, 1976 (Macrouridae: Gadiformes) in Brazilian waters, Southwest Atlantic, using integrative taxonomy

**DOI:** 10.1111/jfb.70401

**Published:** 2026-03-20

**Authors:** Marcelo Roberto Souto de Melo, Flávia Tiemi Masumoto, Heloisa De Cia Caixeta, Marcos Roberto dos Reis Júnior, Claudio Oliveira

**Affiliations:** ^1^ Laboratório de Diversidade, Ecologia e Evolução de Peixes – DEEP Lab, Instituto Oceanográfico Universidade de São Paulo São Paulo Brazil; ^2^ Instituto de Biociências da Universidade Estadual Paulista ‘Júlio de Mesquita Filho’ Botucatu Brazil

**Keywords:** Brazilian economic exclusive zone, continental slope, deep sea, DNA barcoding, grenadier

## Abstract

*Coryphaenoides* Gunnerus, 1765 comprises 66 valid species of deep‐sea fishes commonly known as grenadiers, with 6 previously reported from Brazilian waters. Here, we make the first records for *Coryphaenoides striaturus* and *Coryphaenoides subserrulatus* on the Brazilian continental slope. Both species are distributed in the subtropical regions of the Southern Hemisphere, and the former is being reported for the first time in the Southwest Atlantic, and the latter has its distribution range extended northward. The identifications were confirmed by a unique combination of morphological characters and DNA barcoding using the cytochrome oxidase subunit 1 (COI) gene.

Macrouridae hosts the greatest diversity among the Gadiformes, including more than 300 species in 34 genera, which are commonly known as rattails or grenadiers (English), grenaderos (Spanish) and peixes‐rato or peixes‐rabo‐de‐rato (Portuguese) (Iwamoto, 1990; Nelson et al., 2016). Among these genera, *Coryphaenoides* Gunnerus, 1765 stands out as particularly diverse, including 66 valid species of benthopelagic fishes that inhabit the deep‐sea regions of the world's oceans (Iwamoto, 1990; Fricke et al., 2026). To date, six species of *Coryphaenoides* have been recorded in the Brazilian economic exclusive zone, including *Coryphaenoides leptolepis* Günther, 1877 − the first deep‐sea fish ever described from Brazilian waters − *Coryphaenoides affinis* Günther, 1878, *Coryphaenoides asper* Günther, 1877, *Coryphaenoides mediterraneus* Giglioli, 1893, *Coryphaenoides rudis* Günther, 1878, and *Coryphaenoides thelestomus* Maul, 1951 (Günther, 1877; Iwamoto 1990; Menezes et al., 2003; Melo et al., 2010, 2020).

A recent oceanographic research cruise was conducted onboard the Brazilian NOc *Alpha Crucis* using bottom trawling to evaluate the deep‐sea fish diversity on the continental slope between 200‐ and 1200‐m depth, off Santa Catarina State, Brazil. During that cruise, two previously unreported species of *Coryphaenoides* were collected, *Coryphaenoides striaturus* Barnard, 1925, and *Coryphaenoides subserrulatus* Makushok, 1976 (Figure 1a,b). Soon after collection, the specimens were photographed, and muscular samples were extracted, fixed in 96% ethanol, stored at −20°C and deposited at Coleção Biológica Prof. Edmundo F. Nonato, Instituto Oceanográfico, Universidade de São Paulo (ColBIO). The specimens were fixed in 10% formalin, transferred to 70% ethanol for long‐term preservation and deposited at the Museu de Zoologia da Universidade de São Paulo (MZUSP).

**FIGURE 1:**
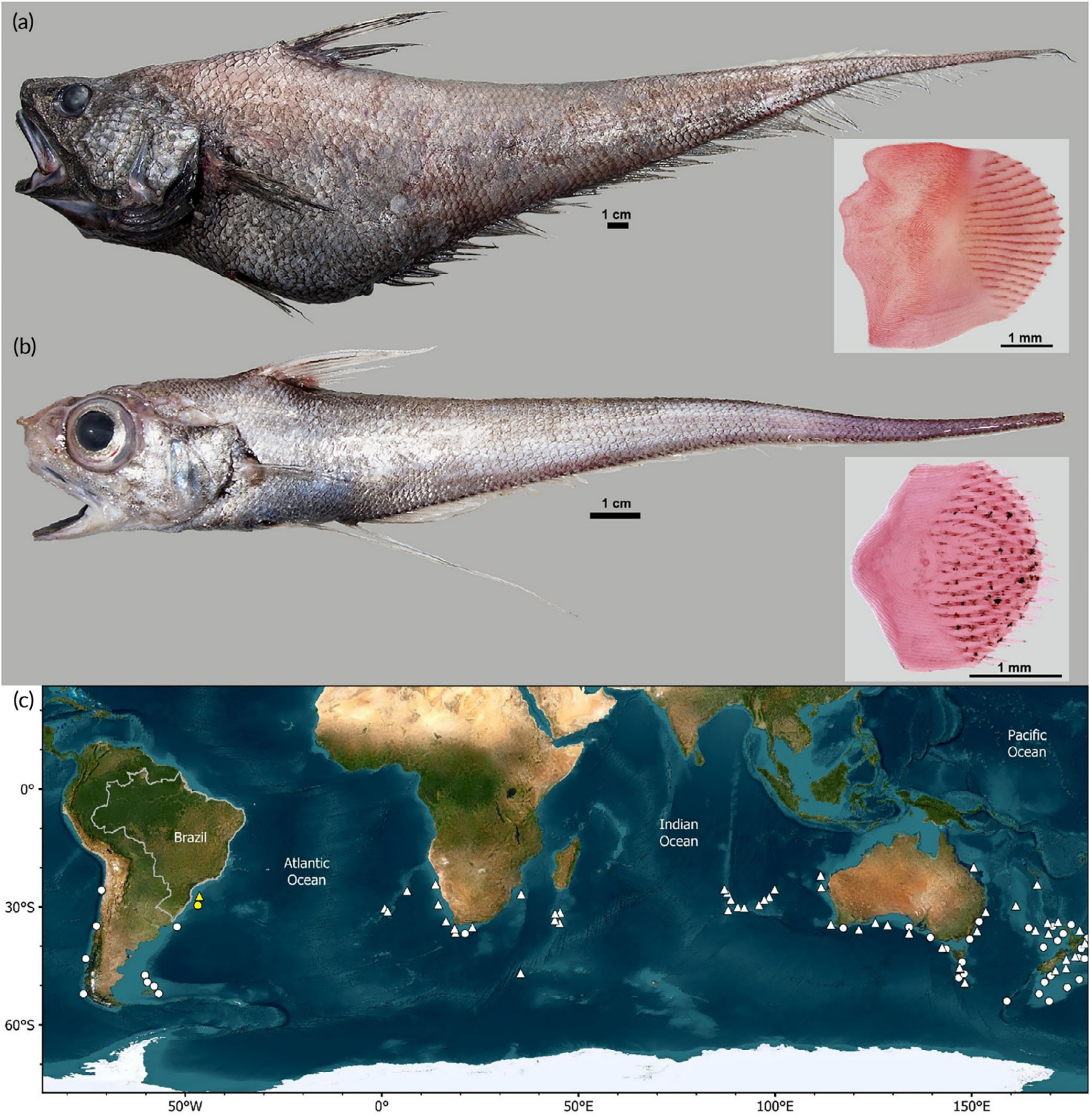
(a) *Coryphaenoides striaturus* in lateral view (scale in detail) [MZUSP 131651, 515.3 mm total length]; (b) *Coryphaenoides subserrulatus* in lateral view (scale in detail) [MZUSP 131649, 214.0 mm total length]; (c) the Southern Hemisphere oceans, with the distribution of *C. striaturus* (triangles) and *C. subserrulatus* (circles) − white symbols represent the previous records (each point may represent more than one locality), and yellow symbols, the new records (records based on GBIF, 2025a, 2025b and double‐checked in Trunov & Konstantinov, 1985; Nakamura et al., 1986; Iwamoto, 1990; Iwamoto & Shcherbachev, 1991; McMillan & Iwamoto, 2015; Pequeño & Vera, 2003; Nión et al., 2016).

Morphometric and meristic data were obtained following Iwamoto (1970) and Iwamoto and Shcherbachev (1988). Measurements were made using a digital calliper to the nearest 0.1 mm. The data presented are based on our specimens in Table 1. Gill‐raker counts are presented from upper lobe to lower lobe. Scales were removed from the body between the lateral line and the first dorsal fin, stained in alizarin red S in 1% potassium hydroxide solution and preserved in glycerin. The sequences of the mitochondrial cytochrome c oxidase subunit I (COI) gene were obtained as described in Caixeta et al. (2024) and compared with sequences available in online databases by BLASTn and BOLD barcode ID tools (Altschul et al., 1990; Ratnasingham & Hebert, 2007). The distribution map was produced using QGIS Association (2025), based on records obtained from GBIF.org (2025a, 2025b), and confronted with literature records.

**TABLE 1:** Morphometric and meristic data for *Coryphaenoides striaturus* and *Coryphaenoides subserrulatus* collected on the southern Brazilian continental slope, Southwest Atlantic.

	*C. striaturus*		*C. subserrulatus*				
			Min.	Max.	Mean	SD	
Measurements							
Total length (mm)	515.3	(1)	144.0	304.6	241.6		(13)
Head length (mm)	96.81	(1)	21.5	62.9	45.5		(12)
Percentage in head length							
Snout length	27.1	(1)	24.6	28.7	26.5	1.3	(12)
Preoral length	11.8	(1)	7.0	14.8	10.4	2.4	(11)
Internasal width	10.5	(1)	6.9	11.0	9.0	1.2	(12)
Posterior nostril length	5.0	(1)	5.7	9.7	7.5	1.1	(12)
Orbit diameter	17.0	(1)	30.0	35.5	32.9	2.0	(12)
Suborbital width	12.9	(1)	7.2	10.3	8.7	1.0	(12)
Postrostral length	79.4	(1)	74.1	78.5	75.8	1.4	(11)
Postorbital length	60.3	(1)	42.2	47.3	45.8	1.6	(8)
Orbit to preopercle length	53.2	(1)	28.6	39.0	32.3	2.7	(11)
Interorbital width	25.9	(1)	22.0	26.4	24.1	1.2	(12)
Upper jaw length	44.3	(1)	38.7	52.3	45.1	3.8	(12)
Barbel length	25.4	(1)	0.8	4.1	1.8	0.9	(12)
First gill‐slit length	18.0	(1)	22.5	29.6	25.1	2.6	(11)
Pre‐anal‐fin distance	185.9	(1)	136.1	164.5	151.8	8.0	(11)
Pelvic‐ to anal‐fin origin length	68.1	(1)	34.2	57.4	43.7	6.2	(11)
Isthmus to anal origin distance	119.9	(1)	95.5	112.7	103.5	5.2	(11)
Isthmus to pelvic distance	58.7	(1)	58.8	79.4	67.0	6.4	(12)
Body depth at 1D origin	118.9	(1)	64.9	83.6	72.6	5.3	(12)
Body depth at anal‐fin origin	100.6	(1)	53.7	65.0	60.1	4.4	(12)
1D to 2D distance	101.1	(1)	4.7	30.8	15.8	7.2	(12)
Body width	53.5	(1)	30.7	41.7	35.2	3.4	(12)
Pre‐1D length	132.2	(1)	107.0	119.2	112.7	3.9	(12)
1D base	33.3	(1)	23.4	35.2	27.1	3.2	(12)
1D height	86.8	(1)	68.4	84.8	73.7	5.8	(6)
Pre‐pelvic‐fin length	129.5	(1)	102.7	134.7	117.6	9.7	(12)
Pelvic‐fin length	51.1	(1)	100.5	169.3	132.8	21.4	11
Pectoral‐fin length	59.5	(1)	77.7	139.9	104.6	19.3	(8)
Counts							
1D rays	II, 8, i	(1)	II, 9 (1); II, 8, i (9); II, 8, ii (2)				
Pectoral‐fin rays	i, 21	(1)	i, 11 (1); i, 12 (3); ii, 13 (1); i, 14 (5); i, 15 (2)				
Pelvic‐fin rays	12	(1)	7 (12)				
Branchiostegal rays	6	(1)	6 (11)				
Outer gill rakers (1st arch)	10	(1)	1,9 (2); 1,10 (2); 1,11 (9)				
Inner gill rakers (1st arch)	12	(1)	2,13 (4); 1,14 (2); 2,14 (5); 2,15 (1)				
Outer gill rakers (2nd arch)	1,13	(1)	2,13 (1); 2,14 (7); 2,15 (4)				
Inner gill rakers (2nd arch)	1,12	(1)	2,11 (1); 2,12 (1); 2,13 (3); 1,14 (3); 2,14 (3)				
Lateral‐line scales	8	(1)	6 (1); 7 (8); 8 (2); 10 (1)				
Scales below 1D origin	7	(1)	6 (1); 7 (5); 8 (2)				
Scales below 2D origin	8	(1)	NA				

*Note*: Number of specimens used inside parenthesis.

Abbreviations: 1D, first dorsal fin; 2D, second dorsal fin; Min., minimum; Max., maximum; SD, standard deviation.

A single specimen of *C. striaturus* was collected [MZUSP 131651, ColBIO‐Mol 1833, 515.3 mm total length (TL), 28°22′35″ S–28°27′39″ S, 46°44′39″ W–46°45′07″ W, 1200 m deep, 05 April 2022]. Among its congeners, *C. striaturus* closely resembles *Coryphaenoides armatus* (Hector, 1875), *Coryphaenoides grahami* Iwamoto & Shcherbachev, 1991, *C. mediterraneus* (Giglioli, 1893) and *Coryphaenoides murrayi* Günther, 1878 by having the upper jaw extending to or beyond the posterior third of the orbit. It can be distinguished from those species by having a fully scaled snout (vs. ventral area of snout unscaled in *C. armatus*, *C. mediterraneus* and *C. murrayi*); pelvic‐fin rays 11–12 (vs. 12–14 in *C. mediterraneus*); interorbital width 23%–30% head length (HL) (vs. 28%–31% HL in *C. grahami* and 28%–34% in *C. murrayi*); long chin barbel 18%–26% HL (vs. 11%–19% HL in *C. armatus*), by lacking a spikelike process on preopercle (vs. present in *C. grahami*), posterior nostril 3%–6% HL (vs. 9% in *C. grahami*) (Iwamoto, 1990; Iwamoto & Shcherbachev, 1991).

Brief description as follows and in Table 1. Trunk short; dorsal gently arched; caudal compressed and greatly elongated, tapering to slender tip and lacking caudal fin; anus slightly posterior to vertical passing through dorsal‐fin base and anterior to mid‐body; belly greatly convex in lateral view. Caudal region compressed and elongated, tapering to a slender tail lacking caudal fin. Head massive, with ridges not apparent; scales covering entire head, including ventral portions of snout, underneath the eye, lower jaw and opercle, but absent from branchiostegal membrane; snout scute absent.

Mouth large and slightly subterminal, upper jaw extending posterior to level of vertical passing on posterior margin of orbit and rictus extending to level of mid orbit. Premaxillary teeth conical, arranged in broad band with external series bearing enlarged and hooked tooth; dentary teeth arranged in a single, irregular row. Two dorsal fins, anterior dorsal elevated in dorsal profile, second spine longest, with anterior margin serrated, lacking filament; posterior dorsal‐fin rays short, extending to caudal tip; anal‐fin origin just posterior to anus, extending to caudal tip; pectoral fin lacking filament; pelvic fin lacking filament. Body scales spinoid, relatively large, not deciduous, armoured with conical spines arranged in parallel rows (Figure 1a).

Pyloric cecum absent (one specimen examined). Bioluminescent organ absent. Ground colour of body and head soon after collection grey, darker on head, belly and ventral caudal portion, upper and lower jaw lips and branchiostegal membrane; anal, dorsal, pectoral and pelvic fins dark with concentrated melanophores; lining of mouth, opercle and branchial cavity pale; parietal peritoneum overall with melanophores concentrated dorsally and ventrally in abdominal cavity, and silver ventrally at pelvic girdle area; visceral peritoneum transparent. Ground colour after preservation grey, with darker regions preserved.

*C. striaturus* was described from off South Africa and is distributed in the Southern Hemisphere, except the Southeast Pacific, between 1190 and 2010 m. It was previously recorded in the Southwest Pacific from New Zealand, Southern Australia, Tasman Sea; in the Indian Ocean from South Africa, West Indian ridge, Madagascar ridge, Walter Shoals, Broken Ridge (west Australian Ridge) and the great Australian Bight; and in the Southeast Atlantic from Namibia, South Africa and the Agulhas plateau (Barnard, 1925; Iwamoto, 1986, 1990; Iwamoto & Shcherbachev, 1991; Iwamoto & Williams, 1999; Trunov, 2001; McMillan & Iwamoto, 2015). This record extends the known species range to the Southwest Atlantic (Figure 1c).

Thirteen specimens of *C. subserrulatus* were collected (MZUSP 131649, ColBIO‐Mol 1193, 2 specimens, 208.9–314.0 mm TL, 28°28′35″ S–28°25′44″ S, 46°48′51″ W–46°49′46″ W, 915 m deep, 29 March 2022; MZUSP 131650, ColBIO‐Mol 1709, 1712, 1759, 1761, 11 specimens, 144.0–304.6 mm TL, 28°24′00″ S–28°19′12″ S, 46°48′26″ W–46°47′25″ W, 985 m deep, 05 April 2025). *C. subserrulatus* can be readily distinguished among its congeners except *C. mcmillani* by having the second pectoral‐fin ray filamentous and elongated, longer than head length (vs. pectoral lacking filamentous rays); and from *C. mcmillani* by having the pelvic‐fin rays 7 (vs. 8, rarely 9), and by the leaflike spines on body scales (vs. needlelike), lighter pigmented body (vs. darker body) and orobranchial cavity dark (vs. pale) (Iwamoto, 1990; Iwamoto & Shcherbachev, 1991).

Brief description as follows and in Table 1. Trunk short; dorsal gently arched; caudal compressed and greatly elongated, tapering to slender tip and lacking caudal fin; anus slightly posterior to vertical passing through dorsal‐fin base and anterior to mid‐body, belly straight in lateral view. Caudal region compressed and elongated, tapering to a slender tail lacking caudal fin. Head delicate, covered by thin skin, with prominent, thin ridges; scales covering the dorsal portion of head and snout, and on infraorbital bones just below eye; scales absent from ventral part of snout, cheek, opercle, branchiostegal rays and membranes, and lower jaw; a stout scute present on tip of snout.

Mouth large and terminal, upper jaw extending posterior to level of vertical passing on posterior margin of orbit and rictus extending to level of mid orbit. Premaxillary teeth minute and conical, arranged in slender band of three or four teeth, and those from external row slightly larger than medial; dentary teeth minute and conical, arranged in slender band of two or three teeth, and those from external row similar to others. Two dorsal fins, anterior dorsal elevated in dorsal profile, second spine longest, with anterior margin serrated and bearing short skin filament; posterior dorsal‐fin rays short, extending to caudal tip; anal‐fin origin just posterior to anus, extending to caudal tip; second pectoral‐fin ray elongated and filamentous; first pelvic‐fin ray elongated and filamentous. Body scales spinoid, small, deciduous, armed with leaf‐like spines not arranged in rows (Figure 1b).

Pyloric cecum absent (one specimen examined). Bioluminescent organ absent. Body and head colour soon after collection silver, darkish on belly, at dorsal midline and ventral midline; dorsal, pectoral and pelvic fins lightly pigmented, dusky with sparse melanophores more concentrated on pelvic‐fin base; anal fin hyaline; lining of mouth, opercle and branchial cavity, and branchiostegal membrane black; parietal peritoneum overall with melanophores concentrated dorsally and ventrally in abdominal cavity, and silver ventrally at pelvic girdle area; visceral peritoneum transparent. Ground colour after preservation beige, with darker regions preserved.

*C. subserrulatus* was described from New Zealand and is distributed in the Southern Hemisphere, between 700 and 1200 m. In the Southeast and Southwest Pacific, it was previously reported from the Tasman Rise and Southern Australia, New Zealand and Chile; in the Indian Ocean, from off South Africa; and in the Southwest Atlantic, from South America around the Falkland/Malvinas islands, Argentina and Uruguay (Bray et al., 2006; Iwamoto, 1986; Iwamoto & Graham, 2001; Iwamoto & Shcherbachev, 1991; Makushok, 1976; McMillan & Iwamoto, 2015; Nión et al., 2016; Pequeño, 1989; Pequeño & Vera, 2003; Trunov & Konstantinov, 1985). This record significantly extends the known species range in the Southwest Atlantic northwards (Figure 1c).

Sequences representing 34 out of 66 valid species of *Coryphaenoides* are available in online databases. The newly generated sequences of both *C. striaturus* (DOP032‐24) and *C. subserrulatus* (DOP033‐24) have 99%–100% similarity with conspecific sequences from the Indo‐Pacific (Tables S1 and S2). The morphological similarities allied to genetic divergences lower than 2% between the Southwestern Atlantic samples and those from other oceans support the conclusion that both *C. striaturus* and *C. subserrulatus* are widely distributed in the Southern Hemisphere (Ward, 2009). This reinforces the need for continued biological sampling to address sampling gaps and improve our understanding of the distribution patterns of deep‐sea fishes.

## AUTHOR CONTRIBUTIONS

Conceptualization: Marcelo Roberto Souto de Melo, Flávia Tiemi Masumoto, Heloisa De Cia Caixeta, Marcos Roberto dos Reis Júnior. Formal analysis: Marcelo Roberto Souto de Melo, Flávia Tiemi Masumoto, Heloisa De Cia Caixeta, Marcos Roberto dos Reis Júnior. Funding acquisition: Marcelo Roberto Souto de Melo, Claudio Oliveira. Investigation: Marcelo Roberto Souto de Melo, Flávia Tiemi Masumoto, Heloisa De Cia Caixeta, Marcos Roberto dos Reis Júnior. Methodology: Marcelo Roberto Souto de Melo, Claudio Oliveira, Flávia Tiemi Masumoto, Heloisa De Cia Caixeta, Marcos Roberto dos Reis Júnior. Marcelo Roberto Souto de Melo. Project administration: Marcelo Roberto Souto de Melo. Software: Marcelo Roberto Souto de Melo, Heloisa De Cia Caixeta. Writing original draft, review, and editing: Marcelo Roberto Souto de Melo, Flávia Tiemi Masumoto, Heloisa De Cia Caixeta, Marcos Roberto dos Reis Júnior.

## FUNDING INFORMATION

This study was financed in part by the Coordenação de Aperfeiçoamento de Pessoal de Nível Superior‐Brasil (CAPES)‐Finance Code 001 (88887.824037/2023‐00 to Flávia Tiemi Masumoto and 88887.644858/202100 to Heloisa De Cia Caixeta, Finance Code 001), Fundação de Amparo à Pesquisa do Estado de São Paulo (FAPESP, 2017/12909‐4 to Marcelo Roberto Souto de Melo; 2020/13433‐6 and 2021/05619‐5 to Claudio Oliveira) and Conselho Nacional de Desenvolvimento Científico e Tecnológico (CNPq 433050/2016‐0 to Marcelo Roberto Souto de Melo, 306054/2006‐0 to Claudio Oliveira, 403380/2022‐7 to Marcos Roberto dos Reis Júnior).

## CONFLICT OF INTEREST STATEMENT

The authors declare no conflicts of interest.

## Supporting information


**Table S1.** Similarity values from barcode ID comparisons of the generated sequence DOP032‐24 with the sequences available on the BOLD Systems database.

**Table S2.** Similarity values from barcode ID comparisons of the generated sequence DOP033‐24 with the sequences available on the BOLD Systems database.

## Data Availability

The data that support the findings of this study are available from the corresponding author upon reasonable request.
